# Subjective Significance Shapes Arousal Effects on Modified Stroop Task Performance: A Duality of Activation Mechanisms Account

**DOI:** 10.3389/fpsyg.2016.00075

**Published:** 2016-02-02

**Authors:** Kamil K. Imbir

**Affiliations:** Faculty of Psychology, University of WarsawWarsaw, Poland

**Keywords:** activation mechanisms, duality of mind, resource competition, duality of activation

## Abstract

Activation mechanisms such as arousal are known to be responsible for slowdown observed in the Emotional Stroop and modified Stroop tasks. Using the duality of mind perspective, we may conclude that both ways of processing information (automatic or controlled) should have their own mechanisms of activation, namely, arousal for an experiential mind, and subjective significance for a rational mind. To investigate the consequences of both, factorial manipulation was prepared. Other factors that influence Stroop task processing such as valence, concreteness, frequency, and word length were controlled. Subjective significance was expected to influence arousal effects. In the first study, the task was to name the color of font for activation charged words. In the second study, activation charged words were, at the same time, combined with an incongruent condition of the classical Stroop task around a fixation point. The task was to indicate the font color for color-meaning words. In both studies, subjective significance was found to shape the arousal impact on performance in terms of the slowdown reduction for words charged with subjective significance.

## Cognitive Control and Duality of Mind

The human mind’s ability to control actions and plan for them in the context of goals and expectations for the future is a milestone in cultural development. Cognitive psychology provides us with a concept of cognitive control (Cooper, 2010; Juvina, 2011) as an easily measurable mind ability that has much in common with our goal realization over time. Recently, the duality of mind approach, which describes and compares two separate mental systems or ways that the mind processes information, namely, automatic and controlled (for review see: Schneider and Chein, 2003), has been gaining attention in the science community (Gawronski and Creighton, 2013). There are several duality of mind theories focusing on specific processes (e.g., persuasion: Petty and Cacioppo, 1986; attitude: Wilson et al., 2000), focusing on a specific domain (such as cognition Strack and Deutsch, 2004) or emotion (Jarymowicz and Imbir, 2015), and, finally, more generally describing mind systems (e.g., Epstein, 2003; Kahneman, 2003, 2011) resulting in different processes. The aim of this paper is to address questions arising from the duality of mind perspective as applied to cognitive control and its probable activation mechanisms.

### Activation Mechanisms Underlying Two Mind Systems

One of the function of emotions is to enhance motivation and action (Frijda, 2007; Kagan, 2007; Damasio, 2010). That is why it is reasonable to search for activation mechanisms specific to each mental system rather than to focus on a single mechanism. Consequently, I distinguish between two activation mechanisms (Imbir, 2015), namely, arousal, specific to the experiential mind, on the one hand, and, subjective significance, specific to the rational mind on the other hand. The reason for distinguishing both types of activation mechanisms is the concept of activation itself. Our mind needs activation mechanisms to sustain motivation to deal with everyday problems. For example, without pleasurable excitation related to exploration of new objects, an organism would not want to get to know such objects, and, consequently, would fail to explore the environment. Psychological research has often demonstrated negative outcomes of activation (meaning arousal) for tasks involving cognitive control and interference control such as the Emotional Stroop Task (EST). But, in fact, such outcomes occur when activation mechanisms are not specific to the task. Duality of mind theory suggests that we should try to discover specific activation for more complex processing. For that reason, dual theories can bring an important contribution to understanding activation mechanisms in both experiential and rational minds.

Arousal is relatively well recognized in psychology. Epstein (2003) argued that arousal level is the factor responsible for shifting the balance between experiential and rational minds toward the former. Arousal can be understood as energy that appears when an organism has to deal with arousing stimuli. This energy activates simple processes, making it easier to run as fast as we can when being chased by a dangerous animal or by another person who wants to rob us. Arousal works on a highly automated level. We do not have to think in order to know that something is threatening our survival or is physically attractive. This recognition immediately comes to mind when we look at it (Kahneman, 2011). Arousal changes associations in our mind by enhancing them for things which are more rather than less arousing. Arousal also influences the quality and results of associations-based processing (Strack and Deutsch, 2014) modifying relations between objects and connections strengthened in the associative store.

Subjective significance is a relatively newly proposed mechanism. It is related to the concept of so-called will power (e.g., Baumeister et al., 1998). To activate and continue careful, energy consuming (Kahneman, 2011), rational and propositionally based (Strack and Deutsch, 2004) thinking, we should engage something which operates at the same level. Arousal is damaging to almost every rational or controlled process because of the shifting balance toward associative mind. Systematic thinking is a luxury (due to the amount of effort required) that, in everyday situations, is better not to use (Kahneman, 2011) lest we overly fatigue ourselves. But why do people engage in such difficult thinking? I argue because they simply want to or think that it is worth doing so. This is probably attributable to the fact that certain situations or ideas may be important from the point of view of one’s goals and expectations for the future. Subjective significance is, thus, a type of attitude toward an object that renders it important and significant, thereby, meriting the investment of energy in accurate systematic processing. Subjective significance could also be referenced to the salience concept (e.g., Kahnt and Tobler, 2013) describing importance of outcomes. For example, in decision making, both gains and losses associated with different options are different in valence but similar in salience. This mean that people perceive them as important in comparison with neutral outcomes that are perceived as non-salient.

**FIGURE 1:**
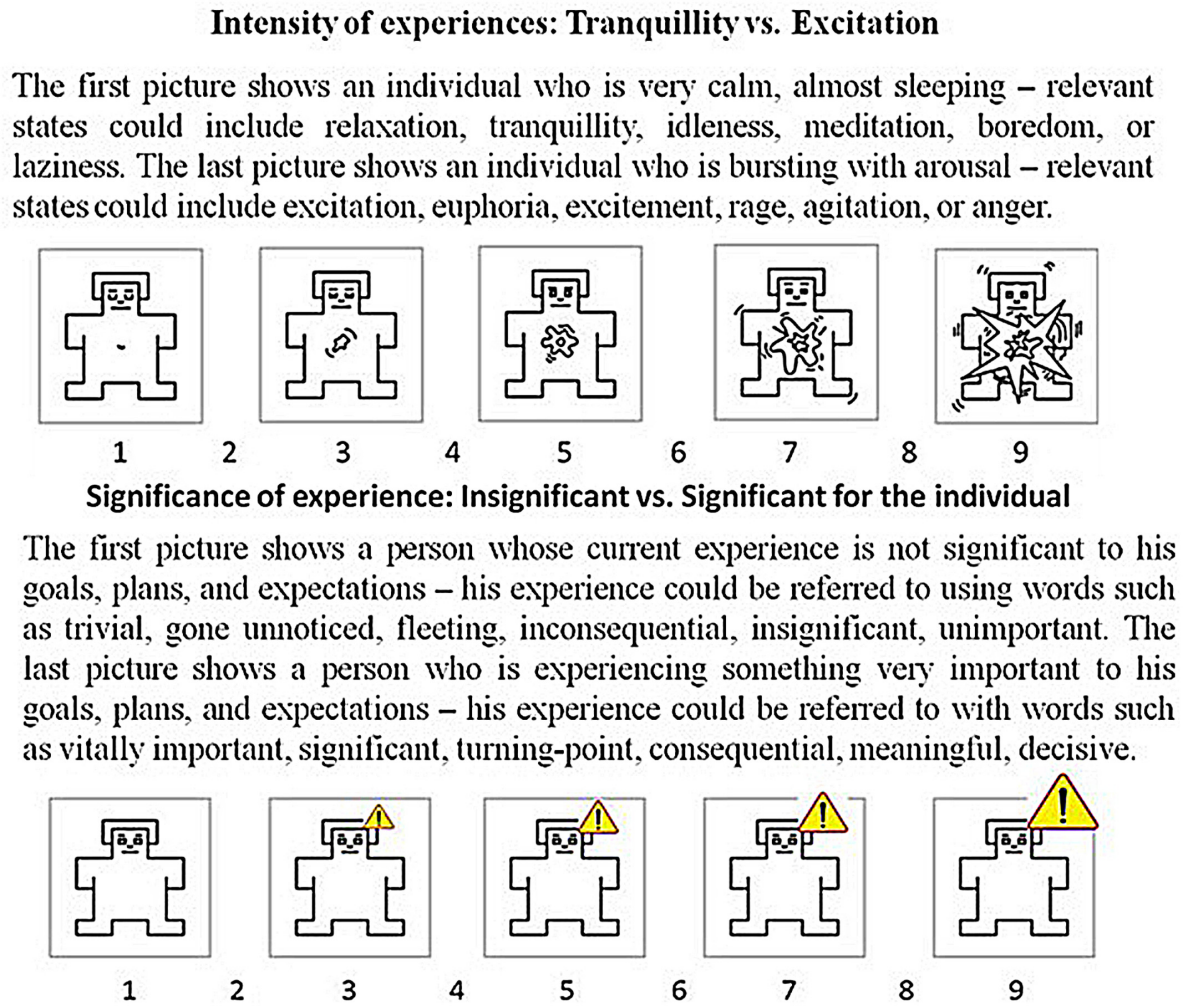
**Self Assessment Manikin (SAM) scales and descriptions used to measure arousal and subjective significance levels of verbal stimuli in ANPW (Imbir, 2015) and ANPW_R (Imbir, submitted)**.

To measure arousal and subjective significance, Self Assessment Manikin (SAM) scales were developed, based on Lang’s (1980) idea of pictorial representation of bodily sensations that do not require a verbal response. This is especially important when arousal is being measured. However, the nature of the rational mind is propositional (Strack and Deutsch, 2004, 2014); thus, to make both scales more comparable, to each was added a description providing context and an explanation of its meaning. Thus, the characteristics of both scales were combined in order to facilitate collection of comparable assessments. **Figure 1** presents both scales used in these normative studies concerning words [Affective Norms for Polish Words (ANPWs): Imbir, 2015].

The ANPW study showed that both scales were reliable in terms of test–retest coherence and split half estimations (c.f. Imbir, 2015). Additionally, arousal and significant assessments were weakly correlated (*r* = 0.24) and, thus, measured different aspects of the activation properties of stimuli. Providing reliable measures of both variables enabled the testing of their contribution to EST processing.

### Cognitive Control and Role of Emotion in Emotional Stroop Task

**FIGURE 2:**
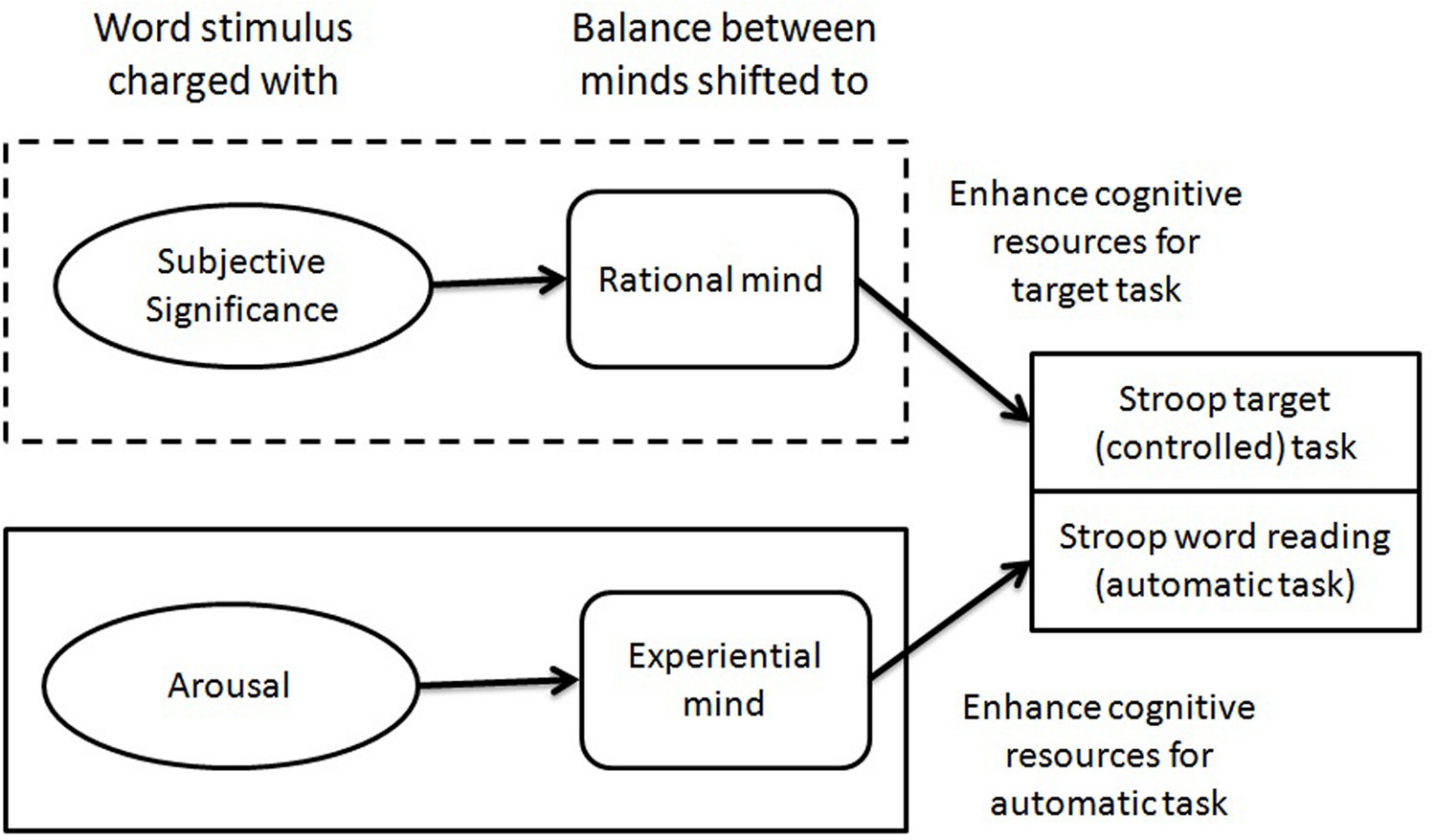
**Duality of mind approach model to Stroop task and predictions for both controlled and automatic parts of Stroop task performance**.

Stroop (1935) introduced a very simple paradigm allowing researchers to measure interference control (Nigg, 2000). The Stroop task is based on a task in which a participant is required to name the color of ink for different words. The task itself generates congruent trials (where the word RED is written in red ink) as well as incongruent trials (where the word GREEN is written in blue ink). The Stroop effects can be observed after subtracting reaction times in congruent trials from those in incongruent trials (Larsen et al., 2006). The difference derives from the interference of the two processes involved in task processing (c.f. **Figure 2**). The first is a controlled and effortful target task (ink color naming) which is rarely performed in everyday experience. The second relates to reading, and semantic access to, content, which, in the case of participants with extensive reading training in school, is highly automated, effortless and even uncontrolled. Both processes may work in the same direction where the probe is congruent or in opposite directions where the trial is incongruent, and where access to semantic meaning, which necessarily requires to be inhibited, gives us the wrong answer. This renders incongruent tests more difficult to perform and thus, reactions take longer.

The EST is slightly different. The task itself is similar to the Stroop task but the trials are different experimental and controlled as opposed to congruent or incongruent. The semantic content of words is especially different, often emotional rather than neutral in meaning (e.g., Williams et al., 1996). EST is very sensitive to the properties of words used (Larsen et al., 2006); thus, when using this paradigm, the words must be carefully chosen for both experimental and control conditions in order to preclude the possibility of irrelevant factors potentially influencing the processing of the task.

There are many studies showing valence effects on EST performance, especially in the case of negatively valenced stimuli (Williams et al., 1996; c.f. McKenna and Sharma, 2004). Some clinical studies involving patients who had suffered traumatic experiences showed that EST slowdown was observed for words connected with traumatic experience (Watts et al., 1986; McKenna and Sharma, 1995, 2004). Apart from valence, EST performance is influenced by at least two important lexical variables. For example, Burt (2002) showed that word frequency influenced the color naming task in terms of response latencies. Less frequently occurring words resulted in longer reaction times as compared with more frequently occurring words. This was probably due to the greater resources required to process the less frequently occurring words and to the capacity of our cognition. Larsen et al. (2006) demonstrated that, among 32 published EST studies, affective words used had lower frequency, longer length, and smaller orthographic neighborhood than the control (neutral) words. They concluded that this could have been the cause of the slowdown reported.

Another important variable is the arousal associated with each valence word used. For example, Dresler et al. (2009), by the careful use of factorial manipulation, showed that the arousal attributable to the word produced emotional interference, independently of valence. Other studies using neuroimaging techniques (fMRI) showed that, in healthy individuals, highly arousing stimuli elicited greater interference than stimuli with low arousal (Compton et al., 2003). Surprisingly this effect was greater for negative than for positive words. All of the above mentioned examples provide evidence that lexical word properties and activation mechanisms play a crucial role in the EST phenomenon; thus such factors must be carefully considered in experimental materials preparation.

Recent studies concerning the emotion duality model impact on cognitive control (Imbir and Jarymowicz, 2013) showed that the types of emotions it tested shaped the EST performance. The automatic emotions-related words (both negatively and positively valenced) generated slowdown in the case of EST as compared with neutral and reflective emotions-related words. This result convinced us to search for the mechanisms underlying cognitive control and duality of mind in order to explain the divergence.

### Duality of Mind in Stroop Task

In the case of both the classical and modified Stroop tasks, two operations are competing for resources. The first one is the explicit task, addressing systematic processing (reflective like), which requires cognitive control to indicate the color of ink in which the word is displayed. The control is required because putting attention on the color of the words is not a spontaneous reaction. To achieve control, a participant must avoid the second process which is highly automated in nature. The reading of words in a visual field is a well-trained skill for any person able to read and practice the skill. Such reading is an excellent example of effortless processing (Kahneman, 2011), characteristic of an automated mind, and gives access to the semantic aspect of a word. In the classical Stroop task, the meaning of the word interferes with the required answer and generates slowdown in reaction times. In EST and modified Stroop task, some aspects of the word (e.g., arousal level or trauma-related content) attract attention and generate slowdown in answering. The dual nature of the Stroop task is expressed in **Figure 2**.

Such construction of Stroop like tasks allows for the measurement of interference control (Nigg, 2000) over automated function of reading and assimilation of semantic meaning. The control itself is an example of effortful processing (Kahneman, 2011) and should, thus, be sensitive to mechanisms characteristic of the rational mind. Previous studies have shown that valence or arousal included in stimuli can make the interference control difficult to maintain; thus, reaction times for controlled tasks are longer. The important question is whether there are some aspects of stimuli that can provide the activation for controlled processing? Taking into account the duality of activation perspective presented in this study, subjective significance may play such a role.

I argue, that arousal of words is the pivotal factor influencing the automated part of Stroop task. More highly arousing words should capture more attention, necessarily meaning that cognitive control should be less effective. But subjective significance should provide the activation for the rational mind. This activation should influence the strength of control, making it easier to give an answer to the explicit task. The difference between arousal and subjective significance is that arousal is in fact a non-verbal, single way dimension (from sleep to excitement: c.f. Russell, 2003), whereas subjective significance is based on a conscious response to stimuli that can be neutral (of moderate subjective significance) but also strong (affirmative of subjective significance) or weak (non-affirmative of subjective significance). The semantic processing during Stroop task trials is associative rather than reflective in nature (c.f. Strack and Deutsch, 2004, 2014). Negation is a reflective operation (Deutsch et al., 2006) requiring time. At the first stage of semantic analysis, there is no time for negation processing; thus, stimuli triggering the significance concept should influence the cognitive control in the same way whether the stimuli be of low or high subjective significance. Only conscious analysis of meaning can trigger a negative association. In **Figure 2**, the duality of activation mechanisms in Stroop task performance is presented with the components influenced by two different aspects of activation.

### Aim and Hypothesis

The aim of the present work was to investigate the activation mechanism underlying the EST effect. Although there is agreement that arousal is the most important factor modulating slowdown for emotional (especially negative) as compared with neutral words, this study sought to examine the duality of activation mechanism predictions. A higher level of slowdown in color naming was expected to be observed in the case of high arousal as compared with low arousal stimuli. It was also expected that subjective significance presence (both low and high level) would modulate this relationship influencing the controlled part of modified Stroop task processing (c.f. **Figure 2**). A moderate level of subjective significance would mean the absence of this factor; thus, in these conditions only an arousal effect would be expected to occur.

## Materials and Methods

### Apparatus

To present stimuli, a standard 15 inch laptop with Windows 7 operating system was used. The experiment script was prepared with E-Prime 2.0 software. Response keys were indicated by stickers with printed symbols: P for orange, C for red, Z for green, and N for blue (which are the first letters of the Polish words describing those colors: *Pomarañczowy, Czerwony, Zielony*, and *Niebieski*). Participants were instructed to use both hands when answering. They were also instructed to keep their fingers over the answer keys at all times during the experiment.

### Materials

To create factorial manipulation, a list of 135 words (nouns) with checked affective qualities were chosen from among 4,905 words. This list was derived from ANPWs Reloaded (Imbir, submitted) which had been compiled using a methodology similar to a previous study concerning affective norms for lower number of words (Imbir, 2015). Two activation dimensions were examined: Arousal and Subjective Significance as well as control affective dimensions such as valence, concreteness and lexical word properties including frequency of appearance in the Polish language (based on Kazojć (2011)) and the number of letters (word length). Assessments for each word used in the current study are presented in Supplementary Materials 1. **Table 1** presents Mean values (*M*) and Standard Deviation (*SD*) for arousal and subjective significance manipulation groups.

**Table 1:** Word properties (*M, SD*) for each manipulation group for experimental conditions (low or high) and control conditions (medium).

		Arousal					
		Low		Medium		High	
Subjective significance		*M*	*SD*	*M*	*SD*	*M*	*SD*
Low	Arousal	3.20	0.29	3.84	0.27	4.79	0.53
	Subjective significance	2.87	0.33	2.96	0.18	2.93	0.60
	Valence	5.25	0.48	5.11	0.44	5.01	0.63
	Concreteness	4.07	1.02	3.88	0.73	3.91	0.90
	LN frequency	6.04	1.86	6.32	1.41	5.95	1.36
	Length	5.73	2.09	6.20	1.32	6.07	2.02
Medium	Arousal	3.20	0.16	3.85	0.26	4.85	0.35
	Subjective significance	3.56	0.32	3.71	0.22	3.74	0.34
	Valence	5.42	0.47	5.38	0.62	5.10	0.66
	Concreteness	3.92	0.90	3.94	0.90	3.98	0.75
	LN frequency	6.50	1.52	6.29	1.58	5.68	1.91
	Length	6.27	2.09	6.13	1.68	6.87	2.45
High	Arousal	3.27	0.27	3.85	0.32	4.97	0.33
	Subjective significance	4.55	0.31	4.64	0.40	4.88	0.44
	Valence	5.38	0.36	5.41	0.35	5.31	1.11
	Concreteness	4.28	0.75	4.33	1.00	4.37	0.98
	LN frequency	6.99	2.02	7.08	1.23	6.55	1.90
	Length	6.47	2.13	6.67	1.95	6.87	2.00

To ensure that words chosen for factorial manipulation were correct, a 3 (arousal levels) × 3 (subjective significance levels) ANOVA was calculated. In the case of arousal ratings, a significant main effect of arousal, *F*(2,126) = 31.09, *p* < 0.001, η^2^ = 0.83, and a no significant main effect of subjective significance, *F*(2,126) = 0.88, *p* = 0.4, η^2^ = 0.01, were found. Taking into account subjective significance ratings, a no significance main effect of arousal, *F*(2,126) = 3.02, *p* = 0.053, η^2^ = 0.04, and a statistically significant main effect of subjective significance, *F*(2,126) = 35.62, *p* < 0.001, η^2^ = 0.81, were found. Although the *p*-value was slightly above 0.05, taking into account the huge differences in η^2^, the factor manipulation was sufficient and independent. In both cases, no interaction effect was found.

To ensure that words chosen for factorial manipulation differed only in the case of manipulated variables, an additional 3 (arousal levels) × 3 (subjective significance levels) ANOVA was run controlling for affective (valence, concreteness) and lexical (natural logarithm of frequency, number of letters) dimensions. In the case of valence ratings, no significant main effect of arousal, *F*(2,126) = 1.46, *p* < 0.23, η^2^ = 0.02, and no significant main effect of subjective significance, *F*(2,126) = 1.89, *p* = 0.16, η^2^ = 0.03, were found. Taking into account concreteness ratings, neither a significant main effect of arousal, *F*(2,126) = 0.03, *p* = 0.97, η^2^ < 0.001 nor an effect of subjective significance, *F*(2,126) = 2.74, *p* = 0.07, η^2^ = 0.04, were found. In the case of frequency estimations, natural logarithm values from Kazojć’s (2011) database concerning the right-skewed distribution (dataset consisted of a number of single word repetitions in a wide range of Polish texts) were analyzed. No significant main effect of arousal, *F*(2,126) = 1.24, *p* = 0.29, η^2^ = 0.02, and no effect of subjective significance, *F*(2,126) = 2.99, *p* = 0.054, η^2^ = 0.05, were found. Finally, word length (number of letters) was assessed and no significant main effect of arousal, *F*(2,126) = 0.57, *p* = 0.57, η^2^ = 0.01, and no effect of subjective significance, *F*(2,126) = 1.29, *p* = 0.28, η^2^ = 0.02, were found.

The above mentioned analyses revealed that the factorial manipulation used enabled the distinguishing between arousal and subjective significance levels of the words used. Furthermore, other factors that could have potentially influenced Stroop task performance such as frequency of appearance, word length, valence and concreteness were controlled. For this reason, observed differences may be attributed only to the designed manipulation.

### Design

A within-subject 3 × 3 factorial design was applied by manipulating word arousal load (Low, Medium, and High) and subjective significance load (Low, Medium, and High). This generated nine groups of words, each containing 15 words. Other factors such as valence, concreteness, frequency of appearances, and numbers of letters in words were controlled and their level aligned between the groups.

Both studies were carried out in accordance with the recommendations of the bioethical committee of the Maria Grzegorzewska University without written informed consent from all subjects. Written consents were not collected as we had assured the participants of anonymity. The oral consent was made by participants in appearance of at least one lab staff member and documented in research diary. This procedure was suggested by the bioethical committee approving research. All subjects gave informed consent in accordance with the Declaration of Helsinki.

### Experiment 1 – Modified Stroop Task

#### Participants

In the first experiment, 60 individuals (30 women) from different Warsaw universities (in equal proportion from the departments of social science, humanities, engineering, life science, and natural science) participated. The sample size of 60 participants was planned in advance. Only correct answers were analyzed; thus, six participants were excluded because they performed poorly and did not provide more than five correct answers in each of the nine conditions. The final analyses included 54 participants (26 women) aged 18–25 years (*M* = 21.13, *SD* = 1.71). All participants were right handed and had normal or corrected to normal (by contact lens or glasses) vision. Before the experiment commenced, the participants were also assessed for normal color vision.

#### Procedure

Before the main experimental session, each participant filled out a socio-demographic questionnaire: age, sex, number of years of education, and academic field of interest. As a training session, each participant performed a standard Stroop task (Stroop, 1935) containing 20 trials (naming color bars squares displayed in one of the four target colors; and reading color meaning words and naming colors of font color-meaning words, both congruent and incongruent). Participants were encouraged to maximize speed of answering and accuracy at the same time. The training session ensured that participants understood the task and how to perform it correctly. Then the modified Stroop test was conducted as an experimental procedure. This test was presented to the participants as a set of 135 trials. Words appeared in a block design in fully random order across each of the nine conditions of the factorial manipulation. The block order was also fully random. A block design was chosen based on evidence showing that EST effects are especially visible in such types of presentations (c.f. Bar-Haim et al., 2007). The task was to indicate the font color in activation charged words. First, a fixation point was presented for a random time from 300 to 600 ms (with 10 ms intervals). This was applied to obviate preparation of expected time range event effects. Then randomly chosen words were displayed in the center of the screen. There was no time limit for response. After choosing the proper letter on the keyboard, the word was replaced by a fixation point (+) for the trial to follow. The words in the entire experiment were presented to participants on a 15 inch monitor in 36 point size, Courier New font using E-Prime 2.0 software. For the entire time words were presented, four letters (P, C, Z, N), indicating possible answers, were displayed on the bottom of the screen.

### Experiment 2 – Combined Stroop Task

#### Participants

In the second experiment, another 60 individuals (30 women) from different Warsaw universities (in equal proportion from the departments of social science, humanities, engineering, life science, and natural science) participated. The sample of 60 participants was planned in advance. Only correct answers were analyzed; thus two participants were excluded because they performed poorly and did not provide more than five correct answers in each of the nine conditions. The final analyses were conducted on 58 participants (29 women) aged 19–26 years (*M* = 21.59, *SD* = 1.76). All participants were right handed and had normal or corrected to normal (by contact lens or glasses) vision. Before the experiment, the participants were assessed for normal color vision.

#### Procedure

As in Experiment 1, participants filled out a socio-demographic questionnaire and then performed a training session with the standard Stroop task for 20 trials based on naming color bars, reading words displayed in black font and naming colors of font color-meaning words which were presented in the center of the screen. Participants were encouraged to work as quickly and accurately as possible as both speed and accuracy were test variables.

**FIGURE 3:**
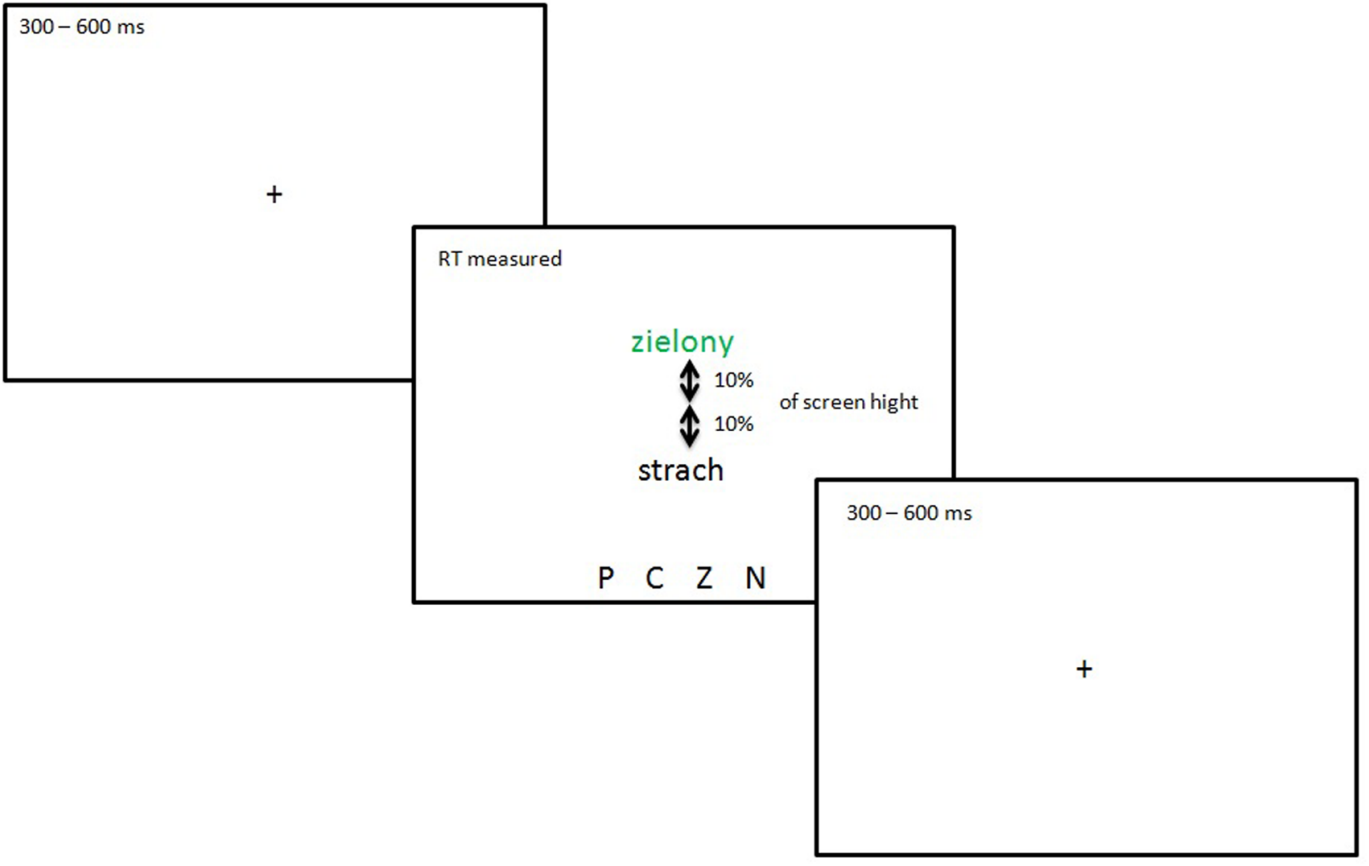
**The combined modified and classical Stroop test prepared on the base of Fackrell et al. (2013) modification used in Experiment 2**.

Following the experimental session, a different version of the modified Stroop task was used. To create it, I used the paradigm modification introduced by Fackrell et al., (2013). This combines classical Stroop task with EST. Target color-meaning words and emotional words were displayed together randomly on the screen 10% higher and lower than the center of the screen. Vertical display was used as Borkenau and Mauer (2006) showed that lateralized presentation elicited asymmetric processing. The task was to indicate the font color of color-meaning words. Each trial was prepared in only mismatch conditions (meaning and color were incongruent). No clue regarding what to do with the other words (displayed simultaneously in black font) was given to the participants. Non-color meaning words were displayed in a block design, with a fully random order of presentation inside each of the nine conditions of the factorial manipulation and sequence of blocks. The experimental session consisted of 135 trials, each timed in the same manner as in Experiment 1. First the fixation point was presented for a random time from 300 to 600 ms (with 10 ms intervals). Then randomly chosen words were displayed above or below the central location. There was no time limit for response. After choosing the proper letter on the keyboard, words were replaced by a fixation point (+) for the next trial. **Figure 3** presents the procedure used in Experiment 2.

The words in the entire experiment were presented to participants on a 15 inch monitor in 36 point size, Courier New font using E-Prime 2.0 software. For the entire time words were presented, four letters (P, C, Z, N) indicating possible answers were displayed on the bottom of the screen.

## Results

To investigate the impact of activation dimensions on performance in both modified Stroop tasks, a repeated measure 3 × 3 ANOVA was computed. Data were aggregated across the conditions for each subject. Each ANOVA was performed on a natural logarithm (LN) of reaction times. This procedure has been widely used in reaction times data and avoids problems with a right-skewed distribution (c.f. Heathcote et al., 1991). In **Table 2**, or in the text, raw reaction times are presented to facilitate better understanding of the observed differences.

**Table 2:** Mean reaction times (in milliseconds) and standard deviations for each of the experimental conditions.

		Arousal		
		Low	Medium	High
		**Experiment 1 – Modified Stroop Task**		

Subjective significance	Low	848 (256)	826 (231)	763 (212)
	Medium	824 (258)	827 (288)	896 (235)
	High	799 (244)	812 (235)	822 (247)

		**Experiment 2 – Combined Stroop Task**		

Subjective significance	Low	1007 (344)	1019 (251)	1015 (283)
	Medium	994 (262)	981 (249)	1084 (251)
	High	1043 (263)	1058 (262)	997 (299)

### Experiment 1 – Modified Stroop Task

The overall error rate in Experiment 1 was 4.92% and all error trials were excluded from further analysis. No significant main effect of arousal, *F*(2,52) = 0.006, *p* = 0.99, η^2^ < 0.001, and no main effect of subjective significance, *F*(2,52) = 1.44, *p* = 0.25, η^2^ = 0.052, were found. A statistically significant interaction effect of arousal and subjective significance was found, *F*(4,50) = 3.21, *p* = 0.02, η^2^ = 0.2. **Table 2** presents mean reaction times and standard deviations for each of the experimental groups.

**FIGURE 4:**
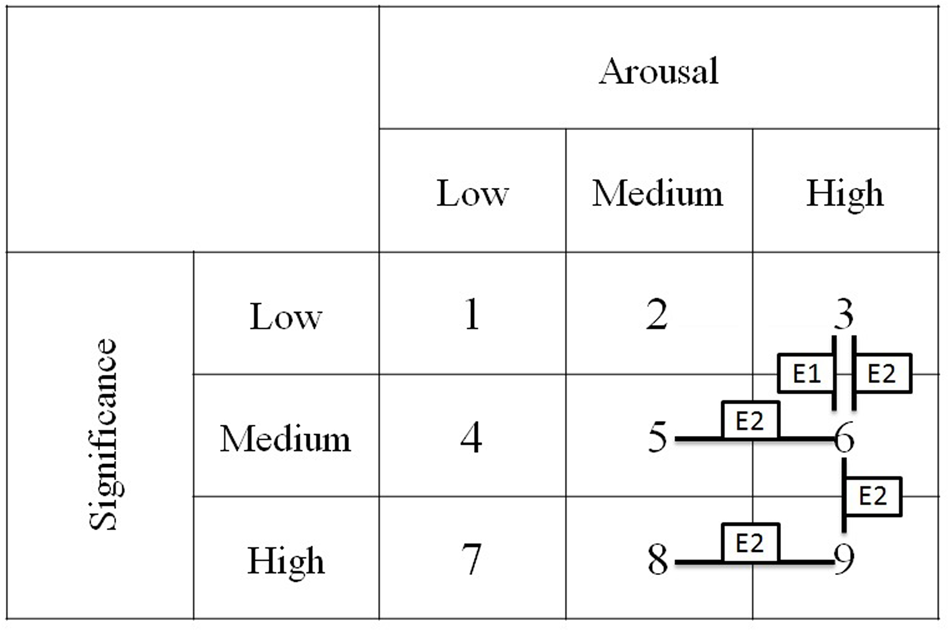
**Pattern of differences observed for experimental groups in both experiments**.

To explore the interaction effect obtained, four additional repeated measures ANOVAs were conducted for each of the subjective significance levels (three analyses of simple main effects of arousal) and for the high arousal level (one analysis of simple main effect of subjective significance). The reason for choosing these simple main effects was based on theoretical expectations connected with the fact that slowdown in reaction times should be observed only for high arousal stimuli. The Holm correction for multiple comparisons was applied. This is a sequentially rejective version of the simple Bonferroni correction for multiple comparisons; thus, one has to divide the critical *p*-value by the number of tests performed at each stage of analysis. In this regard, we may assume that the critical *p*-values were as follows: for the first detected difference *p* < 0.0125 (where four tests were compared); for the second effect *p* < 0.016 (for three performed tests); and, for the third detected difference *p* < 0.025 (for two conducted tests). In each case, the difference contrast was applied to check for effects between low and medium or medium and high manipulating factor groups for each variable. To make the planned analyses more visible, in **Figure 4** one can find experimental design, numbers of manipulation conditions used in text description, and significant contrasts for both experiments.

Taking into account subjective significance simple main effect for high arousal stimuli (groups 3, 6, and 9), a statistically significant difference was found, *F*(2,52) = 6.52, *p* = 0.003, η^2^ = 0.2. Difference contrast analysis showed statistically significant differences between groups of low (3) and medium (6) subjective significance stimuli among high arousal ones, *F*(1,53) = 13.22, *p* = 0.001, η^2^ = 0.2. The remaining difference contrasts were not statistically significant and were not reported.

Taking into account arousal effects, a no statistically significant simple main effect for low subjective significant stimuli (groups 1, 2, and 3) was found, *F*(2,52) = 3.70, *p* = 0.03, η^2^ = 0.13. Difference contrast analysis showed a statistically significant difference between groups of medium (2) and high (3) arousal level stimuli among low subjective significance ones, *F*(1,53) = 6.75, *p* = 0.012, η^2^ = 0.11. No statistically significant simple main effect of arousal either in the case of medium subjective significance stimuli (see groups 4, 5, and 6), *F*(2,52) = 2.53, *p* = 0.09, η^2^ = 0.09, or in the case of high subjective significance stimuli (groups 7, 8, and 9), *F*(2,52) = 0.38, *p* = 0.7, η^2^ = 0.014, were found. No other difference contrast was found to be statistically significant and, thus, were not reported.

### Experiment 2 – Combined Stroop Task

The overall error rate in Experiment 2 was 7.53% and all error trials were excluded from further analysis. Neither a significant main effect of arousal, *F*(2,56) = 0.44, *p* = 0.64, η^2^ = 0.016 nor a main effect of subjective significance, *F*(2,56) = 0.64, *p* = 0.53, η^2^ = 0.022, were found. A statistically significant interaction effect of arousal and subjective significance was found, *F*(4,54) = 4.22, *p* = 0.005, η^2^ = 0.24. **Table 2** presents mean reaction times and standard deviations for each of the manipulation groups in Experiment 2.

To explore interaction effects, four additional repeated measures ANOVAs for each of the subjective significance levels and for high arousal level words were conducted. Holm correction for multiple comparisons was applied. In the case of subjective significance simple main effect in high arousal words (groups 3, 6, and 9) a statistically significant effect was found, *F*(2,56) = 4.51, *p* = 0.015, η^2^ = 0.14. Difference contrast analysis showed statistically significant differences between groups of low (3) and medium (6) subjective significance stimuli, *F*(1,57) = 4.61, *p* = 0.036, η^2^ = 0.08, as well as between groups of medium (6) and high (9) subjective significance stimuli, *F*(1,57) = 4.05, *p* = 0.049, η^2^ = 0.07, among high arousal ones.

Taking into account arousal simple main effects, a no statistically significant effect for low subjective significance stimuli (groups 1, 2, and 3) was found, *F*(2,56) = 0.76, *p* = 0.7, η^2^ = 0.01. A statistically significant effect in the case of medium subjective significance stimuli (see groups 4, 5, and 6) was found, *F*(2,56) = 6.3, *p* = 0.003, η^2^ = 0.18. Difference contrast analysis showed a statistically significant difference between groups of medium (5) and high (6) arousal level stimuli among medium subjective significance ones, *F*(1,57) = 12.43, *p* = 0.001, η^2^ = 0.18. Finally, a no statistically significant effect in the case of high subjective significance stimuli (groups 7, 8, and 9) was found, *F*(2,56) = 2.32, *p* = 0.11, η^2^ = 0.07. Difference contrast analysis showed a statistically significant difference between groups of medium (8) and high (9) arousal level stimuli among high subjective significance ones, *F*(1,57) = 4.58, *p* = 0.037, η^2^ = 0.07. No other difference contrasts were found to be statistically significant and were not reported.

### Additional Analysis of Word Properties

The results obtained suggested checking whether observed results concerning medium and high arousal stimuli could be derived from subtle differences in word properties among each manipulation conditions. To do so, a *t*-test for independent samples taking into account manipulated (arousal and subjective significance) and controlled (concreteness, valence, LN of frequency and length) aspects was conducted. Analyses comparing each of depicted in **Figure 4** as statistically significant contrast effects, namely, as between manipulation conditions 5 and 6, 8 and 9, 3 and 6 as well as 6 and 9 were conducted. For all of these described comparisons, no significant contrasts among controlled variables were found whereas expected significant (*p* < 0.05) contrasts among manipulated variables were found; namely conditions 5 and 6, 8 and 9 differed in the case of arousal ratings whereas conditions 3 and 6, 6 and 9 differed in the case of subjective significance ratings.

## Discussion

This is the first study to combine a duality of mind perspective on activation mechanisms in order to investigate the manner in which arousal and subjective significance shape cognitive control in the case of interference control in the modified Stroop task. Both experiments presented were based on carefully chosen verbal material, contrasting in arousal and subjective significance ratings, but matched in the case of many potentially important variables such as valence, concreteness (c.f. Siakaluk et al., 2014), frequency and length (c.f. Burt, 2002).

In general, no main effects for either variable were found, but an interaction between them was found. In both experiments, differences mostly concerned groups of high arousal words. This pattern of results, perhaps, is due to carefully chosen materials. In some way, this also confirms the validity of proposed factors impacting on Stroop task performance. Simply, it is probable that the main effect may disappear when a new dimension of subjective significance is controlled. An alternative explanation for the lack of arousal effect may be that words included in the lists were in fact moderate arousal ones taking into account a nine-point Likert scale (c.f. **Table 1**). Simply, it is possible that the slowdown could be observed better for higher arousal levels. In the current study, words were chosen in a way that allowed the comparison of three different, increasing levels of arousal, but the highest one was at least moderate. Unfortunately, at this stage, due to both dimensions correlations and correlations between them and controlled variables (Imbir, 2015), it was the only way to prepare a list allowing for the manipulation of both arousal and subjective significance, at the same time as controlling other potentially important factors (valence, concreteness, frequency, and length).

In Experiment 1, slowdown for (relatively) high arousal words was reduced in the low subjective significance group as compared with the moderate group. In Experiment 2, slowdown for (relatively) high arousal words was reduced in both low and high subjective significance groups in comparison with moderate groups and, in both cases, results were statistically significant (c.f. **Figure 4** and **Table 2**). This indicated that the presence of subjective significance factors (low or high) neutralized arousal impact. Subjective significance presence could have influenced cognitive control, motivating and enhancing resources needed for the controlled target task of naming the color of ink (c.f. **Figure 2**). It is interesting that the effect was observed both when the explicit task concerned activation charged words (Experiment 1) and when words were not the subject of the task (Experiment 2). The effects observed in low and high subjective significance groups suggest that the construct of subjective significance is not in fact analogical to unimodal arousal construct, but represents rather bimodal structure (negation and affirmation of subjective significance). The modified Stroop task used in current studies does not allow for the processing of information in a reflective way (Strack and Deutsch, 2014) mostly because quick answers are required and task specificity, namely, the explicit task, is not to read the words, but to ignore their content. For that reason, the results showed no effects of negation. In fact, low and high significant words produced similar outcomes for task performance and lack of significance factor (in moderate groups) resulting in the slowdown observed for high arousal words. Further research is needed to understand the mechanisms of slowdown reduction in a modified Stroop task by subjective significance, but the effects demonstrated in this paper cannot be attributed to mismatching word groups in crucial dimensions such as valence, concreteness, frequency, and length.

Differences in response times in Experiments 1 and 2 were observed. Participants in the first experiment pressed response keys more quickly than in the second experiment. This was due to the nature of the task. In the first experiment, the task was simple because the target stimuli appeared in the center of the screen. In the case of the modified EST, on each occasion, the participants had to find the target stimuli either above or below the fixation point and, thus, had to take more time. The decision to display words in Experiment 2 above or below the fixation point (based on Fackrell et al., 2013) obviated potential lateralization effects demonstrated earlier (c.f. Borkenau and Mauer, 2006).

## Conclusion

The current research showed a new phenomenon concerning Stroop task performance. This was based on a duality of mind approach, which distinguishes between two mechanisms of activation specific to non-verbal experiential system processing and verbalized, rational, and propositional system processing. The Stroop task is a good example of interference between both processes contributing to behavior. Recent findings have shown that valenced or arousal words cause slowdown in response times for ink-color naming task. This study showed that the activation mechanism specific to the controlled part of the Stroop task can reduce arousal level effects.

## Author Contributions

The author confirms being the sole contributor of this work and approved it for publication.

## Conflict of Interest Statement

The author declares that the research was conducted in the absence of any commercial or financial relationships that could be construed as a potential conflict of interest.
