# Supplementary material for: Subjective Significance Shapes Arousal Effects on Modified Stroop Task Performance: A Duality of Activation Mechanisms Account
Source: Front Psychol. 2016 Feb 2;7:75. doi: 10.3389/fpsyg.2016.00075 (PMC4735373; doi:10.3389/fpsyg.2016.00075)
Supplement: Supplementary file 1 [file Data_Sheet_1.DOCX]

Appendix 1. Table present list of all words (in Polish version and translated into English) used to create factorial manipulation (3 arousal levels x 3 significance levels) as well as ratings from ANPW_R - normative study for 4905 Polish words concerning arousal, subjective significance, valence, concreteness and lexical variables such as frequency of appearance based on Kazojć (2011) dataset and length (number of letters).

| Polish word | English translation | Experimental group | arousal category | significance category | Arousal M | Significance M | Valence M | Concreteness M | Frequency of appearance | Number of Letters |
| --- | --- | --- | --- | --- | --- | --- | --- | --- | --- | --- |
| prasowanie | ironing | 1 | 1 | 1 | 3.20 | 3.04 | 4.84 | 3.36 | 23 | 10 |
| seria | series | 1 | 1 | 1 | 3.26 | 2.84 | 5.28 | 4.70 | 1180 | 5 |
| orbitowanie | orbit | 1 | 1 | 1 | 3.90 | 2.70 | 5.22 | 4.72 | 2 | 11 |
| aspekt | aspect | 1 | 1 | 1 | 2.84 | 3.48 | 5.30 | 6.18 | 555 | 6 |
| zero | zero | 1 | 1 | 1 | 3.00 | 3.12 | 4.02 | 4.42 | 1693 | 4 |
| sfinks | sphinx | 1 | 1 | 1 | 3.00 | 2.42 | 5.62 | 3.26 | 176 | 6 |
| echo | echo | 1 | 1 | 1 | 3.42 | 3.14 | 5.64 | 4.08 | 2696 | 4 |
| tenor | tenor | 1 | 1 | 1 | 3.32 | 2.18 | 5.44 | 3.60 | 353 | 5 |
| namiot | tent | 1 | 1 | 1 | 3.56 | 2.92 | 6.24 | 2.04 | 1206 | 6 |
| pauza | pause | 1 | 1 | 1 | 3.08 | 3.12 | 5.04 | 4.92 | 552 | 5 |
| wersja | version | 1 | 1 | 1 | 2.86 | 2.58 | 5.22 | 5.38 | 965 | 6 |
| klan | clan | 1 | 1 | 1 | 3.24 | 2.70 | 5.26 | 3.60 | 539 | 4 |
| pasmo | band | 1 | 1 | 1 | 3.14 | 3.02 | 5.38 | 3.54 | 1104 | 5 |
| smuga | streak | 1 | 1 | 1 | 3.32 | 3.12 | 4.82 | 3.30 | 931 | 5 |
| mila | mile | 1 | 1 | 1 | 2.86 | 2.66 | 5.46 | 3.96 | 781 | 4 |
| loteria | lottery | 2 | 2 | 1 | 4.10 | 3.12 | 5.76 | 3.46 | 56 | 7 |
| imam | imam | 2 | 2 | 1 | 3.59 | 2.85 | 5.00 | 4.14 | 130 | 4 |
| marszałek | marshal | 2 | 2 | 1 | 4.32 | 3.20 | 5.10 | 2.84 | 1315 | 9 |
| gromada | troop | 2 | 2 | 1 | 3.90 | 2.78 | 5.40 | 3.36 | 1175 | 7 |
| sługa | servant | 2 | 2 | 1 | 3.72 | 3.20 | 4.32 | 3.58 | 1668 | 5 |
| akcent | accent | 2 | 2 | 1 | 3.58 | 3.06 | 5.58 | 4.66 | 1076 | 6 |
| wyłom | breach | 2 | 2 | 1 | 4.02 | 3.06 | 4.42 | 3.20 | 278 | 5 |
| plemię | tribe | 2 | 2 | 1 | 3.92 | 3.14 | 5.32 | 3.66 | 1018 | 6 |
| poganin | heathen | 2 | 2 | 1 | 3.66 | 2.94 | 4.64 | 5.10 | 95 | 7 |
| sabat | Sabbath | 2 | 2 | 1 | 4.10 | 2.71 | 4.56 | 5.12 | 913 | 5 |
| olbrzym | giant | 2 | 2 | 1 | 4.04 | 2.70 | 4.98 | 3.24 | 1495 | 7 |
| bufor | buffer | 2 | 2 | 1 | 3.47 | 2.78 | 5.20 | 3.88 | 42 | 5 |
| szlachta | nobility | 2 | 2 | 1 | 4.08 | 2.78 | 5.46 | 3.90 | 811 | 8 |
| hrabia | count | 2 | 2 | 1 | 3.58 | 2.92 | 5.32 | 3.28 | 5345 | 6 |
| odcień | tint | 2 | 2 | 1 | 3.48 | 3.14 | 5.52 | 4.80 | 1024 | 6 |
| galop | gallop | 3 | 3 | 1 | 4.56 | 2.54 | 5.76 | 3.62 | 252 | 5 |
| hazard | gamble | 3 | 3 | 1 | 5.30 | 3.16 | 3.96 | 4.04 | 292 | 6 |
| strzał | shot | 3 | 3 | 1 | 5.04 | 3.94 | 4.56 | 3.14 | 3675 | 6 |
| zombie | zombie | 3 | 3 | 1 | 4.80 | 2.36 | 3.94 | 4.58 | 185 | 6 |
| koszary | barracks | 3 | 3 | 1 | 4.72 | 2.82 | 4.44 | 2.96 | 384 | 7 |
| wolt | volt | 3 | 3 | 1 | 4.31 | 2.41 | 5.08 | 4.44 | 40 | 4 |
| wampir | vampire | 3 | 3 | 1 | 4.90 | 3.00 | 4.52 | 4.42 | 701 | 6 |
| szaleństwo | craze | 3 | 3 | 1 | 6.28 | 4.48 | 5.66 | 6.48 | 2654 | 10 |
| smok | dragon | 3 | 3 | 1 | 4.58 | 2.80 | 5.66 | 4.18 | 3438 | 4 |
| harem | harem | 3 | 3 | 1 | 4.74 | 2.86 | 5.14 | 3.75 | 111 | 5 |
| karykatura | pamphlet / caricature | 3 | 3 | 1 | 4.20 | 2.50 | 5.56 | 3.88 | 131 | 10 |
| parada | parade | 3 | 3 | 1 | 4.08 | 2.76 | 5.64 | 3.42 | 190 | 6 |
| kryminał | thriller / jail | 3 | 3 | 1 | 5.06 | 3.28 | 4.88 | 3.54 | 111 | 8 |
| salwa | salvo | 3 | 3 | 1 | 4.70 | 2.74 | 5.56 | 3.51 | 332 | 5 |
| car | tsar | 3 | 3 | 1 | 4.56 | 2.36 | 4.82 | 2.64 | 1073 | 3 |
| północ | north | 4 | 1 | 2 | 3.37 | 4.16 | 5.76 | 4.68 | 6813 | 6 |
| mgiełka | haze | 4 | 1 | 2 | 2.88 | 3.28 | 5.88 | 2.82 | 494 | 7 |
| kolor | color | 4 | 1 | 2 | 3.40 | 3.70 | 6.04 | 4.88 | 3616 | 5 |
| laik | layman | 4 | 1 | 2 | 3.24 | 3.50 | 4.43 | 5.02 | 52 | 4 |
| arka | ark | 4 | 1 | 2 | 3.08 | 3.32 | 5.74 | 3.08 | 138 | 4 |
| połysk | shine | 4 | 1 | 2 | 3.28 | 3.20 | 5.80 | 3.90 | 407 | 6 |
| fundusz | fund | 4 | 1 | 2 | 3.18 | 3.60 | 5.72 | 4.22 | 308 | 7 |
| kawałek | chunk | 4 | 1 | 2 | 2.94 | 3.52 | 5.16 | 3.65 | 8697 | 7 |
| gamma | gamma | 4 | 1 | 2 | 3.06 | 3.30 | 5.12 | 4.73 | 232 | 5 |
| gleba | soil | 4 | 1 | 2 | 3.18 | 3.34 | 4.94 | 2.36 | 228 | 5 |
| milczenie | silence | 4 | 1 | 2 | 3.26 | 4.31 | 4.60 | 4.92 | 5840 | 9 |
| mieszkaniec | inhabitant | 4 | 1 | 2 | 3.18 | 3.46 | 5.38 | 2.78 | 602 | 11 |
| saga | saga | 4 | 1 | 2 | 3.12 | 3.32 | 5.56 | 4.20 | 770 | 4 |
| trasa | route | 4 | 1 | 2 | 3.42 | 3.84 | 5.50 | 3.04 | 477 | 5 |
| rzemiosło | craft | 4 | 1 | 2 | 3.36 | 3.58 | 5.60 | 4.50 | 336 | 9 |
| chrzest | baptism | 5 | 2 | 2 | 3.74 | 3.78 | 5.72 | 4.90 | 309 | 7 |
| ulewa | downpour | 5 | 2 | 2 | 4.00 | 3.46 | 4.18 | 2.74 | 495 | 5 |
| zaułek | alley | 5 | 2 | 2 | 4.28 | 3.64 | 4.24 | 2.98 | 444 | 6 |
| jazda | ride | 5 | 2 | 2 | 4.20 | 3.62 | 5.82 | 3.44 | 2101 | 5 |
| powieść | novel | 5 | 2 | 2 | 3.50 | 3.96 | 6.08 | 3.88 | 2552 | 7 |
| grupa | group | 5 | 2 | 2 | 3.90 | 3.90 | 5.84 | 3.32 | 5460 | 5 |
| pokaz | show | 5 | 2 | 2 | 3.88 | 3.70 | 5.74 | 4.12 | 1035 | 5 |
| posiadacz | possessor | 5 | 2 | 2 | 3.88 | 3.64 | 5.82 | 4.28 | 212 | 9 |
| interes | business | 5 | 2 | 2 | 4.36 | 3.92 | 5.86 | 4.82 | 3421 | 7 |
| swada | zest | 5 | 2 | 2 | 3.68 | 3.51 | 4.77 | 5.00 | 19 | 5 |
| posag | dowry | 5 | 2 | 2 | 3.68 | 3.26 | 5.58 | 2.71 | 332 | 5 |
| zadatki | smack | 5 | 2 | 2 | 3.80 | 3.68 | 5.32 | 5.34 | 94 | 7 |
| waga | weight | 5 | 2 | 2 | 3.70 | 3.80 | 4.72 | 3.06 | 429 | 4 |
| próba | attempt | 5 | 2 | 2 | 3.76 | 4.12 | 5.30 | 4.90 | 2756 | 5 |
| mrugnięcie | wink | 5 | 2 | 2 | 3.46 | 3.60 | 5.78 | 3.56 | 92 | 10 |
| wojsko | army | 6 | 3 | 2 | 4.96 | 4.16 | 4.94 | 2.90 | 2893 | 6 |
| dziewica | virgin | 6 | 3 | 2 | 5.00 | 4.18 | 6.18 | 3.38 | 446 | 8 |
| labirynt | maze | 6 | 3 | 2 | 4.52 | 3.68 | 5.16 | 3.20 | 1031 | 8 |
| duch | spirit | 6 | 3 | 2 | 4.76 | 3.24 | 4.16 | 5.46 | 5226 | 4 |
| mrok | gloom | 6 | 3 | 2 | 4.48 | 4.06 | 4.18 | 4.24 | 3909 | 4 |
| bieganie | running | 6 | 3 | 2 | 4.66 | 4.04 | 5.94 | 3.24 | 90 | 8 |
| turbo | turbo | 6 | 3 | 2 | 5.06 | 3.78 | 5.76 | 5.33 | 30 | 5 |
| mutacja | mutation | 6 | 3 | 2 | 4.64 | 3.30 | 4.42 | 4.34 | 100 | 7 |
| młodzież | youth | 6 | 3 | 2 | 4.66 | 3.98 | 5.68 | 3.40 | 1703 | 8 |
| promieniowanie | radiation | 6 | 3 | 2 | 4.66 | 3.76 | 4.24 | 4.32 | 705 | 14 |
| sprint | sprint | 6 | 3 | 2 | 4.54 | 3.28 | 5.68 | 3.64 | 38 | 6 |
| wyścig | race | 6 | 3 | 2 | 4.70 | 3.94 | 5.28 | 3.58 | 455 | 6 |
| lesbijka | lesbian | 6 | 3 | 2 | 5.06 | 3.88 | 4.76 | 3.96 | 26 | 8 |
| doping | doping | 6 | 3 | 2 | 5.18 | 3.30 | 5.24 | 4.46 | 23 | 6 |
| orgia | orgy | 6 | 3 | 2 | 5.82 | 3.48 | 4.90 | 4.30 | 108 | 5 |
| poezja | poetry | 7 | 1 | 3 | 2.78 | 4.48 | 6.06 | 5.08 | 955 | 6 |
| wykonywanie | implementing | 7 | 1 | 3 | 3.28 | 4.40 | 5.26 | 4.96 | 243 | 11 |
| krok | step | 7 | 1 | 3 | 2.96 | 4.30 | 5.68 | 3.14 | 12259 | 4 |
| zdanie | sentence/sense | 7 | 1 | 3 | 3.30 | 4.40 | 5.70 | 4.52 | 7177 | 6 |
| osoba | person | 7 | 1 | 3 | 3.72 | 5.16 | 5.82 | 3.72 | 5460 | 5 |
| istota | being | 7 | 1 | 3 | 3.26 | 5.02 | 5.50 | 4.76 | 4054 | 6 |
| godzina | hour | 7 | 1 | 3 | 3.14 | 4.50 | 5.16 | 5.10 | 4687 | 7 |
| singiel | single | 7 | 1 | 3 | 3.84 | 4.10 | 4.88 | 3.74 | 6 | 7 |
| czyn | deed | 7 | 1 | 3 | 3.44 | 5.04 | 5.66 | 4.96 | 1709 | 4 |
| jednostka | unit | 7 | 1 | 3 | 3.00 | 4.60 | 5.02 | 4.08 | 919 | 9 |
| emerytura | pension | 7 | 1 | 3 | 3.42 | 4.36 | 4.90 | 4.18 | 45 | 9 |
| lekcja | lesson | 7 | 1 | 3 | 3.24 | 4.70 | 4.98 | 3.78 | 575 | 6 |
| zasada | rule | 7 | 1 | 3 | 3.16 | 4.58 | 5.40 | 5.38 | 1241 | 6 |
| dokument | document | 7 | 1 | 3 | 3.22 | 4.48 | 5.30 | 3.00 | 1863 | 8 |
| woń | odor | 7 | 1 | 3 | 3.26 | 4.20 | 5.44 | 3.76 | 2810 | 3 |
| wygląd | appearance | 8 | 2 | 3 | 4.32 | 4.66 | 5.82 | 4.58 | 4185 | 6 |
| praca | labor | 8 | 2 | 3 | 4.12 | 5.74 | 5.90 | 4.10 | 6395 | 5 |
| profesor | profesor | 8 | 2 | 3 | 4.14 | 4.74 | 5.86 | 3.16 | 8262 | 8 |
| budżet | budget | 8 | 2 | 3 | 4.30 | 4.34 | 5.32 | 3.48 | 243 | 6 |
| Biblia | Bible | 8 | 2 | 3 | 3.52 | 5.00 | 5.68 | 3.00 | 378 | 6 |
| uwaga | note | 8 | 2 | 3 | 4.42 | 5.02 | 4.88 | 5.60 | 3261 | 5 |
| nawyk | habit | 8 | 2 | 3 | 3.74 | 4.46 | 4.88 | 5.64 | 367 | 5 |
| firma | business | 8 | 2 | 3 | 3.82 | 4.32 | 5.66 | 3.06 | 1505 | 5 |
| szczegół | detail | 8 | 2 | 3 | 3.50 | 4.48 | 5.44 | 4.74 | 1358 | 8 |
| dystans | distance | 8 | 2 | 3 | 3.58 | 4.44 | 4.84 | 4.68 | 1461 | 7 |
| głębia | profundity | 8 | 2 | 3 | 3.74 | 4.36 | 5.26 | 5.48 | 243 | 6 |
| obserwowanie | observation | 8 | 2 | 3 | 3.56 | 4.28 | 5.42 | 4.84 | 219 | 12 |
| wpływ | influence | 8 | 2 | 3 | 3.80 | 4.47 | 5.28 | 5.32 | 3586 | 5 |
| wymiana | exchange | 8 | 2 | 3 | 3.68 | 4.30 | 5.45 | 4.46 | 792 | 7 |
| lekarstwo | medicine | 8 | 2 | 3 | 3.52 | 4.98 | 5.40 | 2.88 | 1126 | 9 |
| geniusz | genius | 9 | 3 | 3 | 4.76 | 5.42 | 7.22 | 5.50 | 936 | 7 |
| poprawka | amendment | 9 | 3 | 3 | 4.52 | 4.46 | 3.90 | 4.16 | 121 | 8 |
| majątek | fortune | 9 | 3 | 3 | 5.04 | 5.28 | 6.54 | 4.02 | 2861 | 7 |
| zwycięzca | winner | 9 | 3 | 3 | 5.56 | 5.86 | 7.42 | 5.24 | 336 | 9 |
| maniak | maniac | 9 | 3 | 3 | 4.92 | 4.34 | 4.30 | 5.20 | 212 | 6 |
| płomień | flame | 9 | 3 | 3 | 4.90 | 4.30 | 5.92 | 3.08 | 2856 | 7 |
| alarm | alert / alarm | 9 | 3 | 3 | 4.48 | 4.80 | 4.16 | 3.42 | 1646 | 5 |
| sesja | session | 9 | 3 | 3 | 5.06 | 4.54 | 3.76 | 4.34 | 253 | 5 |
| ogień | fire | 9 | 3 | 3 | 4.80 | 5.04 | 5.46 | 2.80 | 11105 | 5 |
| buntownik | rebel | 9 | 3 | 3 | 5.54 | 4.86 | 4.80 | 4.24 | 101 | 9 |
| ekstrawertyk | extrovert | 9 | 3 | 3 | 5.17 | 4.59 | 5.28 | 5.64 | 6 | 12 |
| burza | storm | 9 | 3 | 3 | 5.30 | 4.48 | 4.86 | 3.06 | 3238 | 5 |
| wysiłek | effort | 9 | 3 | 3 | 5.14 | 5.10 | 5.26 | 4.52 | 2024 | 7 |
| wynik | result | 9 | 3 | 3 | 4.60 | 5.16 | 5.52 | 4.58 | 1919 | 5 |
| władza | authority | 9 | 3 | 3 | 4.76 | 5.04 | 5.18 | 5.80 | 2056 | 6 |
